# Direct Distillation: A Novel Approach for Efficient Diffusion Model Inference

**DOI:** 10.3390/jimaging11020066

**Published:** 2025-02-19

**Authors:** Zilai Li, Rongkai Zhang

**Affiliations:** 1 Independent Researcher, Guangzhou 510000, China; 2 Division of Control and Instrumentation (CI), Nanyang Technological University, Singapore 639798, Singapore

**Keywords:** computer vision, diffusion distillation, variational information bottleneck, image generative model, multimodal tasks

## Abstract

Diffusion models are among the most common techniques used for image generation, having achieved state-of-the-art performance by implementing auto-regressive algorithms. However, multi-step inference processes are typically slow and require extensive computational resources. To address this issue, we propose the use of an information bottleneck to reschedule inference using a new sampling strategy, which employs a lightweight distilled neural network to map intermediate stages to the final output. This approach reduces the number of iterations and FLOPS required for inference while ensuring the diversity of generated images. A series of validation experiments were conducted involving the COCO dataset as well as the LAION dataset and two proposed distillation models, requiring 57.5 million and 13.5 million parameters, respectively. Results showed that these models were able to bypass 40–50% of the inference steps originally required by a stable U-Net diffusion model, which included 859 million parameters. In the original sampling process, each inference step required 67,749 million multiply–accumulate operations (MACs), while our two distillate models only required 3954 million MACs and 3922 million MACs per inference step. In addition, our distillation algorithm produced a Fréchet inception distance (FID) of 16.75 in eight steps, which was remarkably lower than those of the progressive distillation, adversarial distillation, and DDIM solver algorithms, which produced FID values of 21.0, 30.0, 22.3, and 24.0, respectively. Notably, this process did not require parameters from the original diffusion model to establish a new distillation model prior to training. Information theory was used to further analyze primary bottlenecks in the FID results of existing distillation algorithms, demonstrating that both GANs and typical distillation failed to achieve generative diversity while implicitly studying incorrect posterior probability distributions. Meanwhile, we use information theory to analyze the latest distillation models including LCM-SDXL, SDXL-Turbo, SDXL-Lightning, DMD, and MSD, which reveals the basic reason for the diversity problem confronted by them, and compare those distillation models with our algorithm in the FID and CLIP Score.

## 1. Introduction

Diffusion models [1] are among the most common algorithms used in image generation, having achieved state-of-the-art (SOTA) performance in recent studies [1,2,3]. These algorithms typically include both forward and reverse processes. Specifically, in the forward process, a series of Gaussian noise is iteratively added to the image. The reverse process then employs a neural network to predict the series of noise added in the forward process, denoising the result to generate a high-quality sample. This approach avoids mode collapse problems associated with generative adversarial networks (GANs) [4] by preventing adversarial loss, achieving SOTA quality that is often superior to GANs in image generation tasks. However, the significant computational resources required by the auto-regressive sampling algorithm often limit the application of diffusion methods in real-world tasks. To address this issue, some techniques reduce the cost for each step (i.e., stable diffusion (SD) [5]) by conducting the diffusion step in a latent space, while others minimize the denoising step required by the reverse process using customized sampling methods. Recent studies [6] have demonstrated that the sampling algorithms included in conventional denoising diffusion probabilistic models (DDPMs) [1] can be described by a stochastic differential equation (SDE). There are also ordinary differential equations (ODEs) that describe deterministic transformations and can be used to convert a standard Gaussian distribution into a distribution of images without involving random effects [6]. By considering the sampling process as an algorithm for solving an SDE or ODE, the sampling speed can be accelerated using an existing ODE or SDE solver. For example, the DPM [7] and DPM++ [2] models are specifically designed for differential equations included in the diffusion process. Diffusion distillation is another popular research area for minimizing the sampling step. Computational graphs of the sampling step have also been considered as large neural networks consisting of an identical diffusion model. The resulting massive neural networks have been implemented as teacher models for training smaller neural networks, facilitating the sampling of images with fewer inference steps [8].

Inspired by these techniques, this study systematically combines existing ideas to develop a new distillation algorithm for use in latent space, including a novel sampling time schedule that discretizes ODE solution iterations. More specifically, a composition of the lightweight model (i.e., a non-linear activation-free network (NAFNet) [9]), is used as a block to replace a series of blocks in the original diffusion process. This external lightweight network utilizes intermediate sampling features to directly generate high-quality images. We also employ a sample trajectory provided by the DPM++ solver as the teacher of distillation, proposing a novel sampling time schedule (based on a paper by Karras et al. [10]) to enhance the teacher trajectory. Previous studies [11] have claimed that the choice of method to discretize the sampling trajectory is important for maintaining the quality of generated images. The proposed distillation algorithm was implemented in a latent space provided by stable diffusion to further minimize the computational resources required by the diffusion model. Comprehensive experiments with the COCO dataset [12] and LAION dataset demonstrated that our distillation algorithm could use two concat lightweight models (with 57.7 and 13.5 million parameters) to skip 40–50% of inference steps, which typically require the use of a U-Net with 859 million parameters to calculate stable diffusion results. Each inference step involved 67749 MACs when using original stable diffusion models, while our distillation model only required 3954.12 million and 3922.46 million MACs. Furthermore, our model produced a Fréchet inception distance (FID) of 16.75 during eight-step inference inthe COCO dataset, which was remarkably lower than those of comparable algorithms, such as DDIM [13] (FID of 24.0), standard distillation with various settings [3] (21.0), progressive distillation (30.0), and adversarial distillation [14] (30.0). We also use information theory to analyze FID bottlenecks in existing distillation methods, like LCM-SDXL [15], SDXL-Turbo [16], SDXL-Lightning [14], DMD [17], and MSD [18]. We identify a relationship between GANs and diffusion distillation, both of which implicitly affected the study of posterior probabilistic distributions. Recent research [14,16,17,18] also shows that using adversarial loss in diffusion distillation is a popular area, and we provide a detailed analysis based on our framework to reveal why they confront the diversity problem and how some of it copes with this problem.

Unlike previous methods, which require a student model in the distillation step to be initialized by the original diffusion model, our proposed distillation method does not require knowledge of parameters from the original model. We also further investigate what it means to distill the origin model into the lightweight network that exhibits an entirely different structure to the original stable diffusion model.

## 2. Diffusion Models and Diffusion Distillation

### 2.1. DDPM, Score Matching, and SDEs

Diffusion algorithms can be understood from a variety of perspectives, including one popular interpretation as a special type of variational auto-encoder (VAE) [19]. Diffusion models explicitly include forward and reverse processes that correspond to encoding and decoding steps. In the forward process, Gaussian noise is added step by step to the original image to produce various noisy images, $x_{t}$, during a time step, *t*. For each noisy image, $x_{t-1}$, the following condition holds:

(1) $$p(x_{t}\left|x_{t-1}\right)=N\left(x_{t};\sqrt{1-\beta_{t}}x_{t-1},\beta_{t}I\right),$$

which implies that given the image $x_{t-1}$, the noisy image $x_{t}$ is a Gaussian distribution. Sampling this term involves adding Gaussian noise with a small variance, $\beta_{t}$, to the regressive image $x_{t-1}$, which is obtained by multiplying $x_{t-1}$ with $\sqrt{1-\beta }.$ The choice of $\beta_{t}$ in each time step depends on the different time schedules used in the inference algorithm. The noisy image can then be sampled directly using reparameterization:

(2) $$p(x_{t}\left|x_{0}\right)=N\left(x_{t};\sqrt{{\bar{\alpha }}_{t}}x_{0},\left(1-{\bar{\alpha }}_{t}\right)I\right),$$

in which $\alpha_{t}=1-\beta_{t}$ and ${\bar{\alpha }}_{t}=\prod_{s=1}^{t}\alpha_{s}$. The latter expression can be used to add Gaussian noise directly to $x_{0}$ to sample $x_{t}$, which is equivalent to scaling $x_{t-1}$ and adding Gaussian noise with little variance, β, in Equation (1). The reverse process employs a neural network to study denoising methods, outputting the features $x_{t-1}$ from the input noisy image $x_{t}$. The Bayesian formula then gives

(3) $$p(x_{t-1}\left|x_{0},x_{t}\right)=N\left(x_{t-1};\tilde{\mu_{t}}\left(x_{t},x_{0}\right),\tilde{\beta }I\right),$$

where

(4) $$\tilde{\mu_{t}}\left(x_{t},x_{0}\right)=\frac{\sqrt{{\bar{\alpha }}_{t-1}}\beta_{t}}{1-{\bar{\alpha }}_{t}}x_{0}+\frac{\sqrt{\alpha_{t}}\left(1-{\bar{\alpha }}_{t-1}\right)}{1-{\bar{\alpha }}_{t}}x_{t},$$

and

(5) $${\tilde{\beta }}_{t}=\frac{1-{\bar{\alpha }}_{t-1}}{1-{\bar{\alpha }}_{t}}\beta_{t}.$$

This notation implies that, given $x_{0}$ and $x_{t}$, a less noisy image, $x_{t-1}$, is produced by a Gaussian distribution with a mean of $\mu_{t}$ and a variance of ${\tilde{\beta }}_{t}$. However, during inference, the $x_{0}$ term is agnostic and we only have a noisy image sample, $x_{t}$, from the Gaussian distribution in Equation (2). Previous studies utilizing denoising diffusion probabilistic algorithms (DDPMs) [1] have employed neural networks, with $x_{t}$ used as an input to predict the mean. During training, the neural network predicts various random noise terms added to the original image and studies the mean of the noise, subtracting the average noise from the noisy $x_{t}$ to generate the mean of the next step. In addition, Equation (2) suggests that $x_{t}$ is produced by a Gaussian distribution with a mean proportional to $x_{0}$. For a Gaussian distribution with an unknown mean and only one given sample, this sample can then be directly used as the mean in an unbiased prediction. However, this estimation is overly trivial and previous studies [19] have shown that the best prediction of the mean involves adding a score function to the only sample. Explicitly, the best prediction for the unknown mean μ for the only given sample *z* is then given by

(6) $$E[\mu_{x}\left|z\right]=z+\Sigma_{z}\nabla_{z}\log\left(p_{z}\right).$$

In our case, the score function can be determined using the following [6]:

(7) $$\nabla_{x_{t}}log\left(p_{t}\left(x\right)\right)=\frac{(E_{x_{0}}[p\left(x_{t}\left|x_{0}\right)\nabla_{x_{t}}log\left(p\left(x_{t}\left|x_{0}\right)\right)\right]\right)}{(E_{x_{0}}\left[p\left(x_{t}\left|x_{0}\right)\right]\right)}.$$

The training target in the diffusion model can then be set as

(8) $$\ell =E_{x_{0}}[p\left(x_{t}|x_{0}\right)\left|\right|s_{\theta }\left(x_{t},t\right)-\nabla_{x_{t}}logp\left(x_{t}\left|x_{0}\right)\right|{|}^{2}].$$

Thus, the value of $\nabla_{x_{t}}log(p\left(x_{t}\left|x_{0}\right)\right)$ is essentially the noise added in the original image during the time *t*. The score function is then the average of the noise and using it to sample the image in the next step is equivalent to the sample algorithm propose by DDPM (as discussed above). The framework proposed by Song [6] claims that using a score function to infer a generative image is equivalent to solving an SDE step by step. In our case, we used an existing SDE, given by [7]

(9) $$dx_{t}=\left[f\left(t\right)x_{t}-g^{2}\left(t\right)\nabla_{x}\log q_{t}\left(x_{t}\right)\right]dt+g\left(t\right)d{\bar{w}}_{t},$$

in which

(10) $$f\left(t\right)=\frac{d\log\sqrt{\bar{\alpha_{t}}}}{\;dt},\;g^{2}\left(t\right)=\frac{d{\left(1-\bar{\alpha_{t}}\right)}^{2}}{\;dt}-2\frac{\;d\log\sqrt{\bar{\alpha_{t}}}}{\;dt}{\left(1-\bar{\alpha_{t}}\right)}^{2},$$

where $d{\bar{w}}_{t}$ is random noise from a standard Gaussian distribution and $w_{t}$ corresponds to the standard Wiener process. The optimized target in Equation (8) is also mathematically equivalent to minimizing the evidence low bounce (ELBO) [20] in denoising diffusion probabilistic models [1]. The value of $\nabla_{x_{t}}log(p\left(x_{t}\left|x_{0}\right)\right)$ is essentially the noise added in the original image during the time *t*. The ELBO value introduced by Luo [1] can be expressed as

(11) $$E_{q}\left[D_{\mathrm{KL}}\left(p\left(x_{T}|x_{0}\right)∥q\left(x_{T}\right)\right)+\sum_{t>1}D_{\mathrm{KL}}\left(p\left(x_{t-1}|x_{t},x_{0}\right)∥q_{\theta }\left(x_{t-1}|x_{t}\right)\right)-\log q_{\theta }\left(x_{0}|x_{1}\right)\right].$$

Training targets in the diffusion model are similar to hierarchy VAEs [19], in which various noisy images are actually identical to the different hidden variables in the VAE framework, both of which can be optimized by the ELBO as follows:

(12) $$l=D_{KL}(q_{\theta }\left(x_{1:x}\left|\left(x_{0}\right)\right),p\left(x_{0:x}\right)\right).$$

### 2.2. ODE and DPM++ Solvers

Since the neural network only predicts the mean of the probabilistic distribution of noisy images, $x_{t}$, adding random effects is necessary to ensure the proper sampling of the real features described by Equation (3), which requires a 1000-step inference process to generate high-quality images. Previous studies [6] have identified ordinary differential equations (ODEs) to describe deterministic transformations in which the forward process converts the original probabilistic image distribution into a Gaussian distribution. In this sampling strategy, the probabilistic distribution of each iteration during the forward and reverse processes is equivalent to the distribution described by the SDE in Equation (9). Similarly, an ODE can be used to represent the reverse steps as follows:

(13) $$\frac{dx_{t}}{\;dt}=f\left(t\right)x_{t}-\frac{1}{2}g^{2}\left(t\right)\nabla_{x}\log q_{t}\left(x_{t}\right).$$

This formalism provides us with a methodology for using the score function to iteratively acquire an image without involving random effects, thereby accelerating inference [6] and sampling within 50 steps by directly employing existing ODE solvers. Specifically, DPM [7] and DPM++ [2] are differential equation solvers designed specifically for diffusion processes, which allow for sampling within 20 steps of inference by utilizing the semi-linear structure of the diffusion ODE. The iteration algorithm described by DPM++ is given by

(14) $${\tilde{x}}_{t_{i}}=\frac{\sigma_{t_{i}}}{\sigma_{t_{i-1}}}{\tilde{x}}_{t_{i-1}}+\sigma_{t_{i}}\sum_{n=0}^{k-1}x_{\theta }^{\left(n\right)}\left({\hat{x}}_{\lambda_{t_{i-1}}},\lambda_{t_{i-1}}\right)\int_{\lambda_{t_{i-1}}}^{\lambda_{t_{i}}}e^{\lambda }\frac{{\left(\lambda -\lambda_{t_{i-1}}\right)}^{n}}{n!}d\lambda +O\left(h_{i}^{k+1}\right),$$

where λ describes the signal–noise ratio of individual features.

### 2.3. Time Schedule

**Figure 1:**
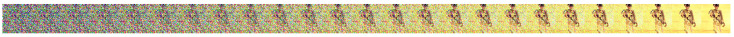
The time schedule proposed by the improved DDPM.

**Figure 2:**
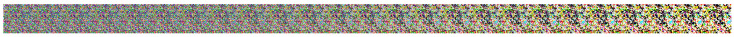
A conventional linear time schedule.

The solutions of diffusion ODEs depend not only on the solver but also on the method used to discretize the process into different intervals during each iteration. As such, different solvers and time schedules produce varying outputs in each step of the ODE process. Previous studies on the improved DDPM [11] have proposed an intuitive time schedule, in which (during the reverse process) noisier regions should be as small as possible, while less noisy areas should be as large as possible. If the truncation errors in diffusion ODEs and SDEs are maintained in regions with the highest noise levels and subsequent steps gradually correct these truncation errors, the quality of generated outputs is likely to improve. Visual results for different time schedules are provided in Figure 1 and Figure 2. Karras et al. [10] suggested that during the denoising process, Δt should gradually decrease along with $\sigma_{t}$ to ensure that truncation errors are mostly concentrated in the high-noise steps. This assumption is justified in their experiment. Specifically, new time schedules offering SOTA results have been developed as follows:

(15) $$\sigma_{i}={\left({\sigma_{max}}^{1/p}+i/\left(N-1\right)\left({\sigma_{min}}^{1/p}+{\sigma_{max}}^{1/p}\right)\right)}^{p},$$

where $t_{i}=\sigma^{-1}\left(\sigma_{t}\right)$ implies utilizing the variance of a distribution, σ, to indicate the time step of the temporary iteration.

### 2.4. Diffusion Distillation

Another method used to accelerate inference involves diffusion distillation, which considers the computational graphs of the recursive ODE solution process to be large neural networks utilized as teacher models to train smaller neural networks, thereby further reducing the number of steps in the diffusion model generation process. Prevalent distillation methods include progressive distillation [21], guide distillation [3], consistency distillation [22], and knowledge distillation [8]. The first two methods require the model to predict the output of the next step to accelerate the inference process, while the others predict the final output directly in the first step.

While training diffusion models from scratch typically requires substantial computation quantities, the distillation process requires relatively little time and considerably fewer resources compared to training a new generative model. For convenience, parameters in the distillation networks are often initialized to match those of the original diffusion model. However, initialization by a well-trained network is not the only factor affecting the reduction of computational costs. Typically, when training a diffusion model, the particular noise added to the image, $x_{0}$ in Equation (2), is known, but the value of the final target ($\nabla_{x_{t}}log\left(p\left(x_{t}\right)\right)$) is not known before training, since it requires a value for the average amount of image noise when only the original image and corresponding noise are given. The distillation algorithm can thus utilize a pre-trained diffusion model to acquire this score function, which reduces the required training resources. This study also demonstrates that diffusion distillation can still increase training speed, even when a simple model is randomly initialized.

## 3. A New Distillation Algorithm for Diffusion Models

### 3.1. An Improved Karras Time Schedule

Conventional time scheduling used in diffusion models suggests that noisier sections should be as short as possible during inference [11], while less noisy sections should be as large as possible. It is evident from the results shown in Figure 1 and Figure 2 that using the noisy image in the first few steps to directly generate a final image resembles sampling an image with a hidden variable provided by the distribution in β-VAE [23] with a KL term less than the normal VAE used. The conventional VAE training experience [23,24] suggests that reducing the KL term constraint allows the latent variable distribution to deviate from a standard Gaussian distribution to a low-dimensional feature similar to the final output, which facilitates the acquisition of higher-quality decoding images [23]. In extreme cases, when the KL term constraint is zero, this is simply an ordinary auto-encoder. Hence, it is intuitive to use a small neural network to generate noisy hidden variables that do not require high-precision results, since it is typically assumed that a well-trained generative model (e.g., β-VAE) would output a similar image with small disturbance to the input. Then, a complex neural network should be applied, which corresponds to a large number of iterations, using an identical diffusion model to generate the final image. Recent research [25] also mathematically proves that adding disturbed σ into the input *x* will not change the output of the diffusion model beyond O(σ). Actually, the original DDPM algorithm adds noise in each step of training and inferring.

Hence, if the perturbed input is sampled from the same probability distribution as the original input, the image quality remains unaffected since the model has generalization capability. Experimental results from an improved DDPM [11] confirmed the advantages of this intuitive strategy, which uses a large neural network to fine-tune noisy input and generate high-quality images. The study by Karras [10] furthered this theory by proposing a more generalized noise scheduling method. In the paper, the time step *t* was replaced by $\sigma_{t}$ for convenience during the diffusion process (providing a more concise formula for deduction). A new noise variance schedule was then introduced in which the variance was uniformly divided in an exponential noise space using Equation (8). As *p* increased, variance terms closer to 0 were divided more finely, allowing the time step to be arbitrarily stretched toward the time step at 0.

**Figure 3:**
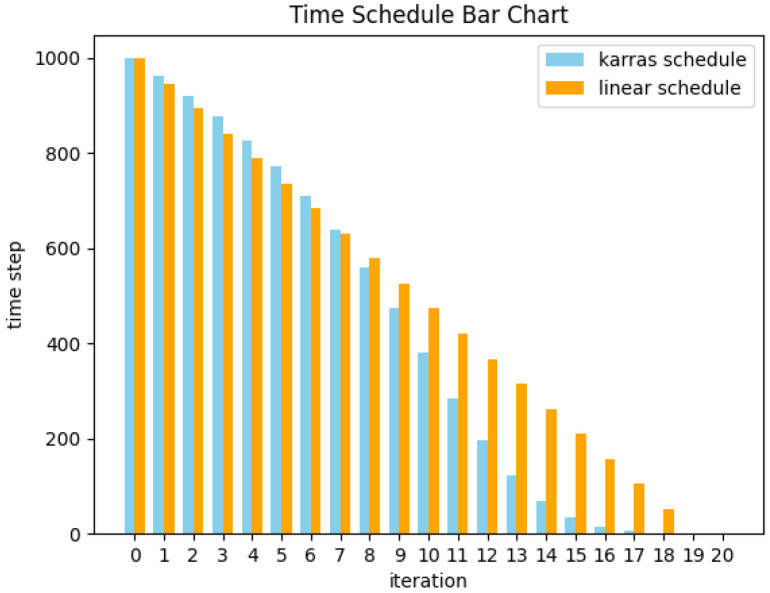
Various time steps in the time schedule developed by Karras and a linear time schedule.

Figure 3 demonstrates that in regions where feature noise levels are relatively high, the interval between each step *t* is smaller than the linear schedule. A simple solution to this issue involves performing uniform division in exponential time-space as follows:

(16) $$t_{i}={\left({t_{max}}^{1/p}+i/(N-1)\left({t_{min}}^{1/p}+{t_{max}}^{1/p}\right)\right)}^{p}.$$

**Figure 4:**
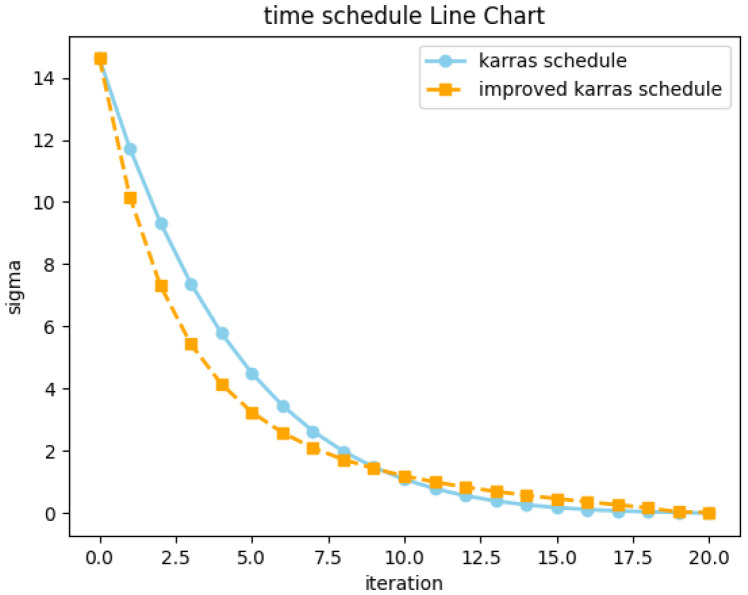
Variance between the proposed schedule and the Karras schedule.

In this case, as *p* increases, Δt for time steps closer to the final step *T* becomes larger. This approach also offers other benefits. For example, in the Karras time schedule, the final sections are prevented from being divided too finely in a 20-step denoising process. As seen in Figure 3, the fourth-to-last step in the Karras schedule corresponds to the sixth of 1000 steps in the diffusion model. The last two steps correspond to less than one step out of 1000, which is insignificant considering the denoising capabilities of the diffusion model. In contrast, Figure 4 demonstrates that the third-to-last step corresponds to the 31st out of 1000 steps. This noise term grows more slowly in the lower noise levels until it quickly approaches the Karras curve as *t* increases. This accelerating time schedule allows the noise level to match that of Karras in the final forward step, as shown in Figure 4. Experimental results confirmed that with 20 steps, the images generated by our time schedule exhibited better FID values than images generated using the Karras method. Previous results have indicated that while diffusion models can generate high-quality images with 10 inference steps, 20 steps are typically needed to successfully generate converged images [10]. As such, the proposed time schedule is highly practical for a variety of real-world generation tasks.

### 3.2. Direct Distillation in Latent Space

This paper proposes a distillation algorithm to accelerate the sampling process, in which a series of feature pairs, $\left(x_{0},x_{t}\right),t\in \left\{T/2+\beta -\epsilon ,T/2+\beta -\epsilon +1,\ldots ,T/2+\beta +\epsilon \right\}$, are sampled in the reverse process generated by the diffusion model and used as supervised data. The basic method proposed by Luhman [8] is then used to optimize targets and train a distillation model, which can output a final result using an input from intermediate features (in a reverse process) as follows:

(17) $$\begin{array}{cc} L_{\mathrm{student}\;}= & E_{x_{t},t}[D_{KL}(p_{\mathrm{teacher}\;}\left(x_{0}|x_{t}\right)\\ & \;\;\;\;\;\;\;∥p_{\mathrm{student}\;}\left(x_{0}|x_{t}\right))]. \end{array}$$

**Figure 5:**
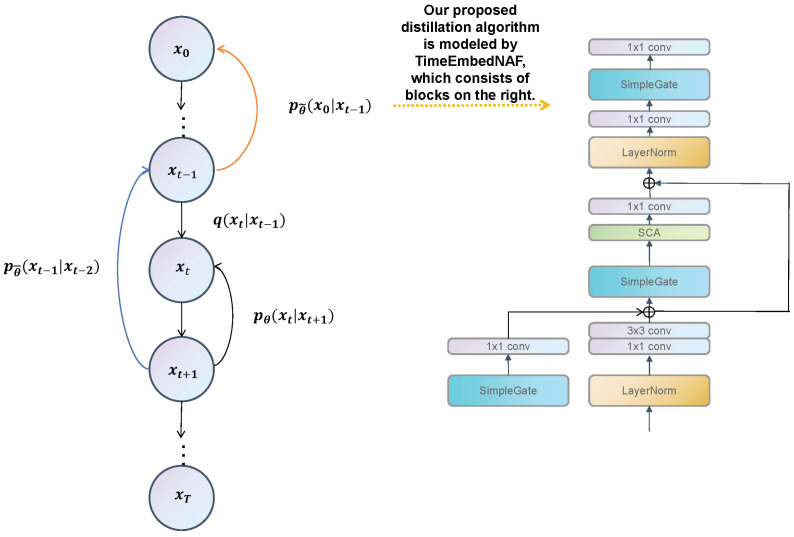
The underlying concepts of direct distillation, in which the orange line denotes the proposed distillation process, the blue line represents progressive distillation, and the black line denotes the normal diffusion process. The $p_{\theta }(x_{t-1}\left|x_{t}\right)$ term models the reverse process, while $q(x_{t}\left|x_{t-1}\right)$ models the forward process.

This target can be optimized by simply minimizing the L2 distance between outputs from the distillation step and the original model. However, considering the similarity between our distillation and denoising tasks, using an empirical technique for denoising allows the loss function to be reasonably set as follows [26]:

(18) $$L=E_{x_{0},x_{t}}\left[d\left(x_{0},f_{\theta }\left(x_{t},t\right)\right)\right],$$

where d() is an arbitrary distance (i.e., normal L1 or L2 loss) and $f_{\theta }$ is the distillation model parameterized by θ. Experiments verified that this algorithm, under the guidance of the information bottleneck framework, ensured the diversity of generative images for producing better FID results than conventional distillation methods [3,8,21]. Additional details are provided in Figure 5, which demonstrates the basic concepts of distillation.

It is intuitive that shifting the correct score function from pointing in the ${\hat{x}}_{0}\left(x_{t},t\right)$ direction to pointing in the ${\hat{x}}_{0}\left(x_{t-1},t-1\right)$ direction (with the output in the next step [3,21]) or to pointing in the final output ($x_{0}$) direction [22] enhances the mutual information of features and outputs, since it shifts the output feature from the mean of a series of images to the particular output result. A precise proof for this step will be discussed in this section. The information for the bottleneck framework used in the generative model [27] can then be described as follows:

(19) $$\ell_{IB}=I\left(Z;G\left(Z\right)|\theta \right)-\beta I\left(Z;G\left(Z\right)|\theta \right),$$

where I(z;G(Z)) describes the mutual information of the features and output. This expression is rational as it provides a trade-off between the mutual information of the output and intermediate features, thus preventing the model from learning by rote memorization [28]. A trade-off with mutual information (MI) allows detailed features to be output in a concise way as information passes the bottleneck. However, MI can be difficult to calculate, and the variational information bottleneck (VIB) [29] compensates for this difficulty by considering the upper bounce of the MI as follows:

(20) $$I\left(x;z\right)\leq E_{x}[D_{KL}(p_{\theta }(z\left|x\right)\left||q\left(z\right)\right],$$

in which q(z) is a standard Gaussian distribution. The diffusion model denoising process is then a Markove chain. We also considered the data process inequality (DPI) introduced by Tishby [28], which describes the characteristics of MI in an arbitrary Markove chain (a->b->c). In this Markove chain, I(A,C) is always less than I(A,B); this gives

(21) $$\begin{array}{c} I(X_{0},{\hat{X}}_{\theta ,t-1}\left({\hat{X}}_{\theta ,t},t\right)\left|\theta \right)\leq I({\hat{X}}_{\theta ,0}\left({\hat{X}}_{\theta ,t-1},t-1\right),{\hat{X}}_{\theta ,t-1}\left({\hat{X}}_{\theta ,t},t\right)\left|\theta \right)\\ \;\leq E_{{\hat{x}}_{\theta ,t-1}}[D_{KL}(p_{\theta }\left({\hat{x}}_{\theta ,0}\left({\hat{x}}_{\theta ,t-1},t-1\right)\right)\left|{\hat{x}}_{\theta ,t-1}\left({\hat{x}}_{\theta ,t},t\right)\right)\\ \;\left||q\left({\hat{x}}_{\theta ,0}\left({\hat{x}}_{\theta ,t-1},t-1\right)\right)\right)], \end{array}$$

where ${\hat{x}}_{\theta ,0}$ is the $x_{0}$ value predicted by the neural network and ${\hat{x}}_{\theta ,t-1}$ is a noisy image input to layer t−1. The final results of this formula are only variable for the mean and variance of $p_{\theta }\left({\hat{x}}_{\theta ,0}\left(x_{t-1},t-1\right)\right)\left|{\hat{x}}_{\theta ,t-1}\left(x_{t},t\right)\right)$. Notably, the mean increases when shifted from the original output to the final output in a given distillation, like progressive distillation [3,21]. In addition, the variance of possible model output during training does not increase, since the variance of the studied output probability decreases in each subsequent step. The resulting increase in MI between intermediate features and the final training output can limit model generalizability [27]. Similarly, Kawaguchi et al. [30] demonstrated that mutual information determines the upper bounds of generalization errors. The basic idea of the paper [30] is that the generalization ability is ensured by the capacity of the model. For an arbitrary dataset, *S*, if the capacity of the model is limited, then enough observed data can ensure the model approaches the best hypothesis of observed data that the model can have. It also claims that the distance between the output of the average hypothesis it can obtain and a particular hypothesis from a trained model is bounced, and this means that the performance of the particular model can output good results for various data points. And, the paper [30] claims that, although the maximum number of hypotheses a model can have is $2^{n}$ for a dataset, *S*, with *n* data points, the amount of typical input data points is only $2^{H(X)}$, where H(X) is the entropy of the variable *X*. To further constrain the capacity of a particular model training on SGD, we can consider the probability distribution of the feature *z* given the input *x*, where p(z|x) explicitly denotes the random map between x and z, and the volume of *X* encoded in *Z* is $2^{H(X|Z)}$. Hence, all possible *Z* taht a model can have is $2^{I(X,Z)}=\frac{2^{H(X)}}{2^{H(X|Z)}}$, which constrains the number of possible hypotheses of a model.

Prevalent distillation [3] also produced generative images with obviously higher quality than the normal diffusion model in an eight-step inference process, though the FID scores were similar to those of the normal diffusion model [3]. Recent research in diffusion distillation can also be analyzed in this framework. Some distillation algorithms [16,17] use GANs and adversarial loss to directly match Gaussian noise and the corresponding images generated by the teacher model. Those distillation algorithms can generate high-quality images while confronting the bottleneck problem of FID. Explicitly, the papers [3,16,17] showed that the latest generative image samples from LCM-SDXL [15], SDXL-Turbo [16], SDXL-Lightning [14], and progressive distillation [3] had better image quality, while their FIDs were 22.16, 23.24, and 24.46 respectively, which were similar to that of the original diffusion model using a DDIM sampler whose FID performance was 24.0. Meanwhile, the research [18] proposes a distillation algorithm that trains multiple one-step generators as student models to predict different subset images directly, since this can prevent the capacity limit of the one-step model from becoming the bottleneck of the FID performance. In the framework of our analysis, training a one-step generator and shifting the score function from a blurred image to an image in the typical set, like progressive distillation, would increase the mutual information between output images and input features, hence causing diversity problems.

Diversity problems associated with GANs can also be solved using a method similar to our algorithm [27], which offers a trade-off between input and output in the mutual information, as discussed in the Section 3.3. We will further analyze the relationship between GANs and diffusion distillation.

### 3.3. A Mathematical Analysis of Diversity Problems in Both Distillation and GAN Models

The score function provided by diffusion models, a special type of VAE [19], estimates the original image of a given noisy image via the standard to minimize the MSE between the ground truth and the prediction. Hence, the resulting image tends to be the mean of images that can generate the noisy input image during the diffusion process. Noisy images in the diffusion model correspond to the hidden variable *z* in the original VAE. However, in most distillation algorithms, a shift occurs from the mean of images to a typical image, explicitly enhancing the mutual information between the output and features during training. For example, progressive distillation shifts this direction to the mean image which originally outputs in the subsequent step to increase the inference speed, hence enhancing the mutual information between features and outputs. Similarly, normal VAE models tend to generate blurred images using the hidden variable *z* as an input, while GANs directly generate typical images, implicitly preventing a study of the mean of p(x|z) to prevent blurring. It has also been shown that, under the guidance of information bottleneck theory, adding a neural network at the beginning of an InfoGAN-like generator can transform the inputted Gaussian noise into another Gaussian noise [27]. This approach minimizes MI between the generator input and output, which is beneficial for enhancing the FID and is similar to our proposed algorithm, offering a trade-off between the MI of outputs and features in the initial layer.

Specifically, differences in GAN and VAE performance are explicitly analyzed in Bayesian form and provide insights into why our algorithm works to ensure diversity. The β-VAE term is also shown to be a special type of auto-encoder obeying VIB [23,29]. Further analysis shows that the ELBO optimized by β-VAE consists of two parts, which optimize $p_{\theta }\left(x\right)$ while allowing $q_{\phi }(z\left|x\right)$ to approach $p_{\theta }(z\left|x\right)$, explicitly indicating that $q_{\phi }(z\left|x\right)$ exhibits maximized entropy. However, a GAN [31] without an explicit design may fail to approximate the true value of $p_{\theta }(z\left|x\right)$, causing mode collapse [32]. More precisely, the VAE loss function can be equivalently rewritten in a Maximum A Posteriori (MAP) form by involving constants that are unrelated to the optimization:

(22) $$L\propto \log(\underset{p_{1}=p(x_{0:n}\left|z,\phi \right)}{\underset{︸}{\frac{e^{-\beta_{1}*E_{x}[CrossEntropy(q_{\phi }\left(z\left|x\right),p_{\theta }\left(x\left|z\right)\right)\right]}}{Z_{1}}}}*\underset{p_{2}=p\left(\phi ,z\right)}{\underset{︸}{E_{x}[\overset{p=p(\phi ,z|x)}{\overset{︷}{\frac{e^{-\beta_{2}*D_{KL}(q_{\phi }(z\left|x\right)\left||p\left(z\right)\right)}}{Z_{2}}]}}}}),$$

In the following discussion, we use abbreviations to provide a concise description, where $E_{1}=E_{x}[CrossEntropy(q_{\phi }\left(z\left|x\right),p_{\theta }\left(x\left|z\right)\right)\right]$; $E_{2}=D_{KL}(q_{\phi }\left(z\left|x\right)\right|\left|p\left(z\right)\right)$; and $p_{1}$ and $p_{2}$ denote Boltzmann distributions.

Our aim for this discussion is to introduce a simplified scenario that describes the tendency for updates in *z* and ϕ during optimization. The Plato allegory [19] claims that the hidden variable *z* can be considered a necessary characteristic of the observable output image, while the VAE encoder studies this proper hidden variable. We then focus on training and the ways in which this hidden variable affects the generative diversity. During this discussion, the decoder used for training is considered ideal, which can be examined using the ELBO of VAE as follows:

(23) $$\ell =\underset{normal\;AE\;loss}{\underset{︸}{\int -q_{\phi }(z\left|x\right)logp_{\theta }(x\left|z\right)dx}}+\underset{KL\;constrain}{\underset{︸}{\int -q_{\phi }(z\left|x\right)log\frac{p(z)}{q_{\phi }(z\left|x\right)}dx}},$$

in which normal AE loss allows the outputted hidden variable *z* to be similar to the image *x*, while the KL term allows the hidden variable to be sampled from a standard Gaussian distribution. When the decoder is ideal, the hidden variable converges to the expected value in the Plato allegory.

In Equation (22), $p_{\theta }(x\left|z\right)$ is a probability distribution provided by an ideal decoder. The energy $E_{1}$ is an objective function that determines whether the encoder properly parameterizes probabilistic distributions of *z* when generating high-quality *x* terms, since optimizing the cross entropy implies maximizing the log-likelihood to generate *x* in the original VAE. With constraints on the maximum entropy criteria and observable energy results denoted by $E_{1}$, the Boltzmann distribution $p_{1}$ provides an unbiased prediction of $E_{1}$, which considers $p_{1}$ to represent the likelihood of training images $x_{0:n}$ in a batch with temporary choices for ϕ and *z* during the minimization of $E_{1}$. Similarly, $p_{2}$ denotes the prior probability of choosing ϕ and *z*, which forces the neural network to model *z* as similarly to a standard Gaussian distribution as possible. Hence, the ELBO *L*, optimized by the VAE, is proportional to the posterior distribution of p(ϕ,z|x), assuming that p(x) is a constant. Experiments [23] have shown the images generated by VAEs are often blurred, which means if the assumption that p(x|z) is a Gaussian distribution is correct, the blur produced by the VAE is caused by high variance in the output distribution. We also assume that for two normal neural networks using different loss functions, given similar network structures, parameters, and hidden variables, the probability distribution for training images is a Gaussian distribution with high variance:

(24) $$p_{\theta }(x\left|z\right)=N\left(x;G_{\theta }\left(z\right),\sigma I\right).$$

The GAN loss may then automatically allow the model to learn a biased output and prevent the loss caused by studying the mean of high variance distribution. More explicitly,

(25) $$p_{\theta }\left(G\left(z\right)\right)=\int p_{\theta }(G\left(z\right)\left|z\right)p\left(z\right)dz,$$

with *z* determined from a standard Gaussian distribution. The $p_{\theta }\left(G\left(z\right)\right)$ terms also differ from the real p(x) and cause the probability $p_{\theta }\left(z\right)$ to shift from a standard Gaussian distribution:

(26) $$p_{\theta }\left(z\right)=\int p_{\theta }(z\left|x\right)p\left(x\right)dx,$$

where *x* is from real image probabilities p(x) and the $p_{\theta }\left(z\right)$ may not follow a Gaussian distribution. Thus, rectifying $p_{\theta }\left(z\right)$ could prevent mode collapse, as observed in previous studies [32]. In that paper, a neural network studying the p(z|G(z)) observed that $p_{\theta }\left(z\right)$ did not follow a Gaussian distribution and could be used as a signal to rectify the GAN, which meant that in the area of mode collapse, $p_{\theta }\left(z\right)$ had a higher probability than the real input distribution and hence could only generate real images when the input *z* in this area was at a frequency higher than the real input’s frequency. As such, we develop a simplified mathematical proof to provide further insights.

As such, we first consider a GAN loss function [31]:

(27) $$\begin{array}{cc} \max_{\omega }\min_{\theta }\ell_{\mathrm{GAN}}\left(\omega ,\gamma \right) & :=E_{z}\left[\log\sigma \left(D_{\omega }\left(G_{\gamma }\left(z\right)\right)\right)\right]\\ & +E_{x}\left[\log\left(1-\sigma \left(D_{\omega }\left(x\right)\right)\right)\right], \end{array}$$

in which $G_{\theta }$ is the generator and $D_{\omega }$ is a discriminator used to determine whether the output is from the real dataset. If the discriminator determines that outputting a constant score to a special class of images can minimize the loss, the gradient will disappear and make training the generator impossible [32], which will also affect p(z|θ). We explicitly formulate the gradient update process as a Markov chain in which temporary values of θ depend on previous $\hat{\theta }$, the input image *x*, and the parameter ω in the discriminator. As such, p(z|θ) is the distribution of *z* depending purely on the temporary parameter θ and input *x*, a direct consequence of being the latest element in the Markov chain following θ. Hence, for p(z|θ,x), we have

(28) $$\begin{array}{c} p(z\left|\theta ,\omega ,\hat{\theta },x\right)=p(z\left|\theta ,x\right)=\frac{p_{\omega }(\theta \left|z,\hat{\theta },x\right)*p(z\left|\hat{\theta },\omega ,x\right)}{p_{\omega }(\theta \left|\hat{\theta },x\right)}, \end{array}$$

and

(29) $$\begin{array}{c} p(z\left|\omega ,\hat{\theta },x\right)=p(z\left|\hat{\theta },x\right) \end{array}$$

in which $\hat{\theta }$ is a given parameter prior to optimization and ω is a set of discriminator parameters that define the generator gradient. In the case of $p_{\omega }(\theta \left|\hat{\theta },x\right)$, we follow previous work [33], which assumes that parameters obey the Gaussian distribution when using L2 regularization. The latent variable *z* should then generate an image in the mode collapse region. As a result, $p_{\omega }(\theta \left|z,\hat{\theta },x\right)$ is close to 1, since the gradient would not update θ in this situation and would force the p(z|θ,x) term to be high during each iteration. Hence, if we assume that for each possible image, *x*, the probability of it is an identical constant, we have the bias $p_{\theta }\left(z\right)$. A similar result was observed by Srivastava et al. [32], who noted that, given an image in a mode collapse region, the real prior probability of *z* was higher than the real probability. This suggests that generating a real output distribution requires the probability of the input to be higher than that of the real input in the mode collapse area.

Similarly, an information bottleneck-based training algorithm was proposed by Jeon et al. [27] to address issues with mode collapse. This framework simply includes a module at the beginning of an Info-GAN-like generator to transform the input hidden variable *z* into standard Gaussian noise. This unique Info-Gan generator first enhances the mutual information of an inputted hidden variable, *z*, and outputs an image, *x*. A network is then forced to project *z* onto another standard Gaussian noise sample, *r*, which serves as an input to the generator and automatically regresses the mutual information. In contrast, parts of the distillation algorithm shift the middle features pointing to the mean of a series of images to the final output result, thus enhancing the MI between the middle feature and the output (prevented by our proposed algorithm). As in the Bayesian analysis mentioned above, we assume that the IB-GAN transforms from *z* to *r* to acquire a proper distribution, $p_{\theta }\left(z\right)$, in Equation (26) that is similar to a standard Gaussian distribution to prevent diversity issues. In contrast, although not directly mentioned in information bottleneck theory, recent SOTA progressive distillation [14] further utilizes adversarial loss to enhance the image quality of distillation models but fails to maintain diversity in progressive distillation [3]. Other researches [16,17] also show that using adversarial loss and GANs to distill diffusion model is a popular research topic while confronting the bottleneck of FID performance. To explicitly explain how the bias of posterior distribution affects the mutual information, we have

(30) $$\begin{array}{c} p(x\left|z,\theta \right)=\frac{p(z|\theta ,x)p(x)p(\theta )}{p(z)p(\theta )}, \end{array}$$

in which inputted Gaussian noise, *z*, and ground truth image, *x*, are sampled from a real distribution and independent of θ; hence, studying a biased posterior distribution will affect the mutual information and generalizability.

### 3.4. Non-Linear Activation Free Network for Diffusion Distillation

Unlike other distillation methods, our student model was not initialized with a diffusion model. Instead, we employed a customized non-linear activation-free network (NAFNet) as the student model was used to learn Equation (11). Compared to stable diffusion, our parameter counts and computational requirements were significantly lower than those of a U-Net in stable diffusion, resulting in faster training and higher inference speeds. The model was randomly initialized at the start of training. In the case of features with varying degrees of noise, the same ground truth was learned, effectively treating the process as a simple denoising task. The practicality of our proposed algorithm was limited by the computational resources required for adding a new diffusion model during inference. The noise level of our input was equal to the fifth-from-last input in a 20-step linear schedule, which allowed for the use of a lightweight network in studying the output. Convergence was accelerated following a process proposed by Zhao et al. [26], in which L1 loss was used for five-sixths of the data during training, with MSE loss used for the remaining data. However, given the relatively short time required for each offline distillation iteration, this method had a minimal impact on the overall distillation technique. A customized NAFNet also allowed the input at a specific time, *t*, to be used as auxiliary information for determining corresponding noise levels (see Figure 5). Each input feature then corresponded to a time step, *t*, in the diffusion process. Experimental results showed that our network produced satisfactory outputs for the noisy feature input without any prompts, achieving a PSNR of more than 30 dB. As such, we did not include a prompt-processing mechanism to further streamline the network. As previously mentioned, time embedding was used to customize NAFNet blocks, as illustrated in Figure 5. In other words, once our U-Net received a time step input, it was directly converted into a standard time embedding, as proposed by transformer studies [34]. The result was then passed through a linear, fully connected layer, and the output was fed into the activation function. The output was subsequently passed through another linear, fully connected layer to obtain the time embedding, which was then passed to each block in subsequent layers. Each block within the U-Net was equipped with a specific module used to process the time embedding. However, unlike in the diffusion model, all activation functions in our network were based on a NAFNet simple gate, a purely linear activation function that offers a non-linearity similar to a GELU. The significantly smaller parameter sizes in NAFNet, compared to in the stable diffusion model U-Net, allowed for the stacking of multiple instances of NAFNet to enhance performance.

## 4. Experiment

We first compare performance differences between the proposed time schedule and the Karras time schedule used in the COCO dataset [12]. Specifically, we compare the FID [35] for images generated by the stable diffusion model and the DPM++2m ODE solver for different time schedules, using images generated by the Karras time schedule in 12 steps as a baseline for comparison with images generated by our distillation algorithm. Experiments showed that the FID results for our new time schedule in 20 steps were slightly better than those of the Karras time schedule. The proposed distillation algorithm also offered similar FID performance for normal samples with stable diffusion in 12 steps. We further compare our 7+1-step distillation to other prevalent progressive distillation algorithms, observing that the 8-step FID performance achieved by our algorithm was superior.

### 4.1. Time Schedule

We randomly sampled 10,000 captions from the COCO caption validation set and implemented stable diffusion using two different time schedules and the sampler described in Table 1. Generated images were then compared with images corresponding to the captions used to calculate FID values. The results showed that our time schedule outperformed the Karras time schedule, as confirmed by the FID values shown in Table 1. Previous studies [2] have shown that although diffusion models can generate high-quality images within 15 steps, images generated in 20 steps offer better convergence, thereby providing a viable choice for real-world tasks.

### 4.2. Distillation Algorithm

During the training of the distillation model, we directly sampled 40,000 captions from the COCO dataset and generated 40,000 corresponding images. We then extracted output features from the 10th, 11th, and 12th steps of the diffusion process, generating 120,000 intermediate features. Of these, 100,000 features were used as the training set, while the others formed the test set, with the corresponding final output features from the diffusion process serving as the ground truth.

The training step was conducted in two stages. In the first stage, the distillation step was treated as a simple denoising task. The model was then trained using the COCO dataset, learning to output the ground truth from intermediate features. The feature size was 4 × 64 × 64 and training was conducted with a batch size of 64 for 60,000 iterations using the Adam [36] optimizer ($\beta_{1}=\beta_{2}=0.9$; a weight decay of 0). The original learning rate was set to 0.001 and a cosine annealing time schedule [37] was included with 36 blocks in the TimeEmbedNAFNet and a depth of 3.

Training could then be treated as a denoising task [26], in which L1 loss could be used as an optimized target to accelerate convergence, with L2 loss used to further minimize the loss term. We then implemented a second-stage distillation approach, with experimental observations revealing that some texture details in the generated images were lacking. As such, we aimed to further enhance these image details with deblurring methods. Specifically, in the second training phase, we utilized a NAFNet with 36 blocks, concentrating the block counts in the shallow decoder layers to further deblur features and improve details. Training settings were the same as before, with only a simple L2 loss used as the objective function. The parameter amount as well as the computational cost in inference are shown in Table 2.

The final results indicate that when using the COCO caption validation set as the ground truth, there were only two-point differences in the FID between the output of our 12-step distillation and the output from the new schedule. Additional details are provided in Table 3 and Table 4, in which we compare high-quality image generation with an inference step larger than eight steps, which is one of the popular choices for distillation models. We also compare outputted results from the distillation model with those of stable diffusion using the Karras time schedule. The validation set for this comparison included captions from a random selection of 10,000 images in the COCO caption validation set, with results shown in Table 5.

**Figure 6:**
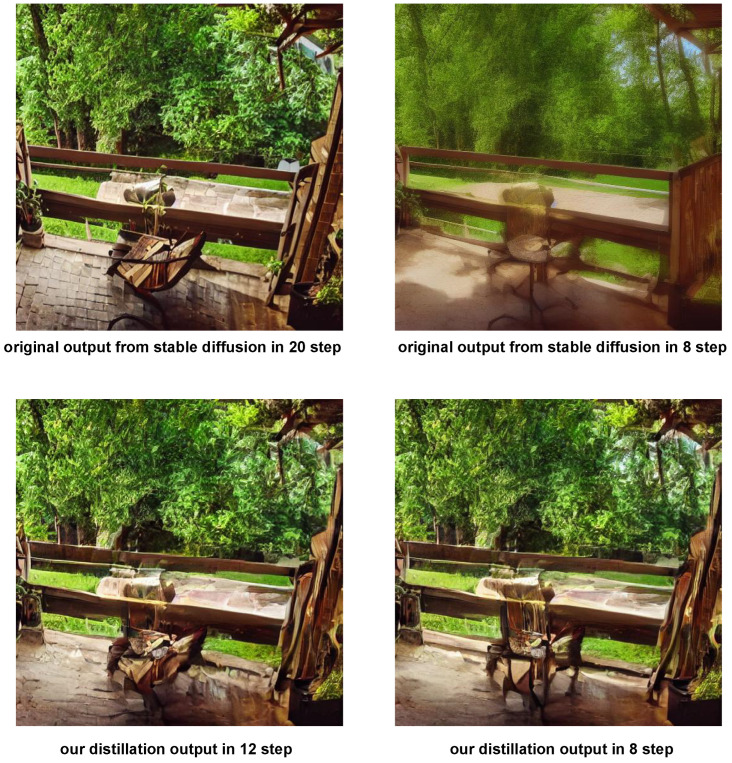
Output images from the original stable diffusion and our novel distillation algorithm.

**Figure A1:**
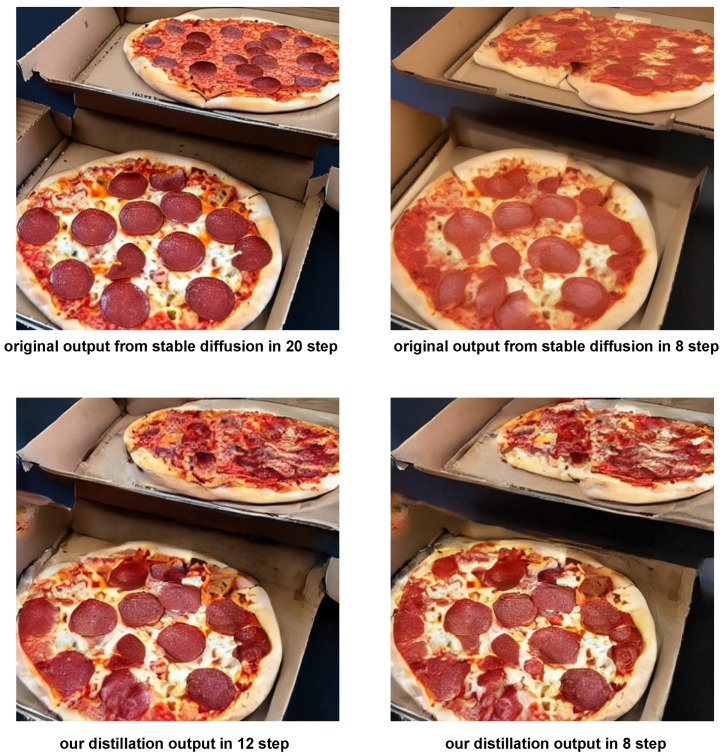
Output images from the original stable diffusion model and our novel distillation algorithm.

**Figure A2:**
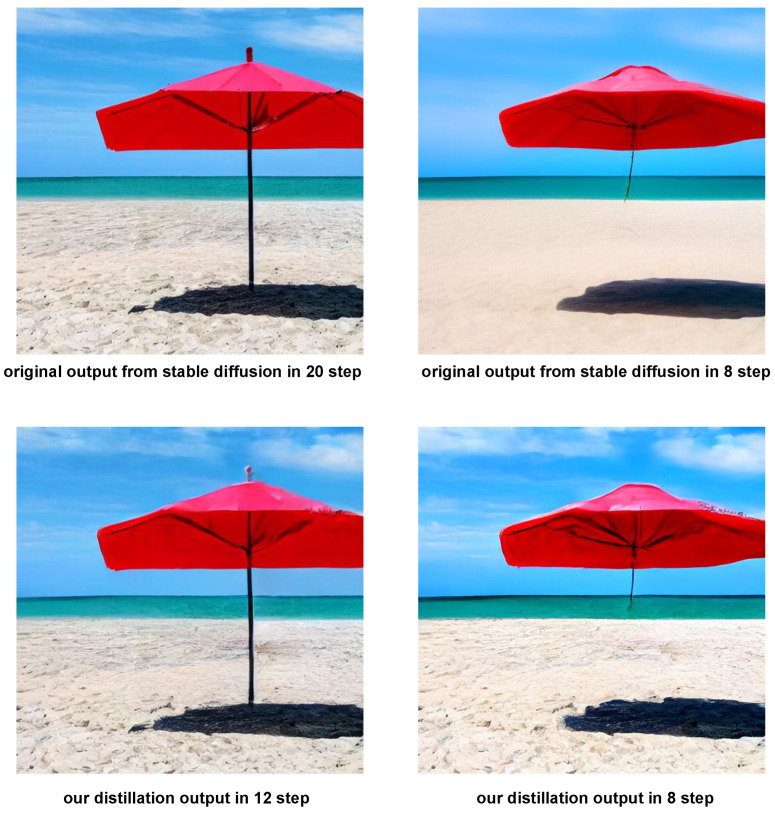
Output images from the original stable diffusion model and our novel distillation algorithm.

**Figure A3:**
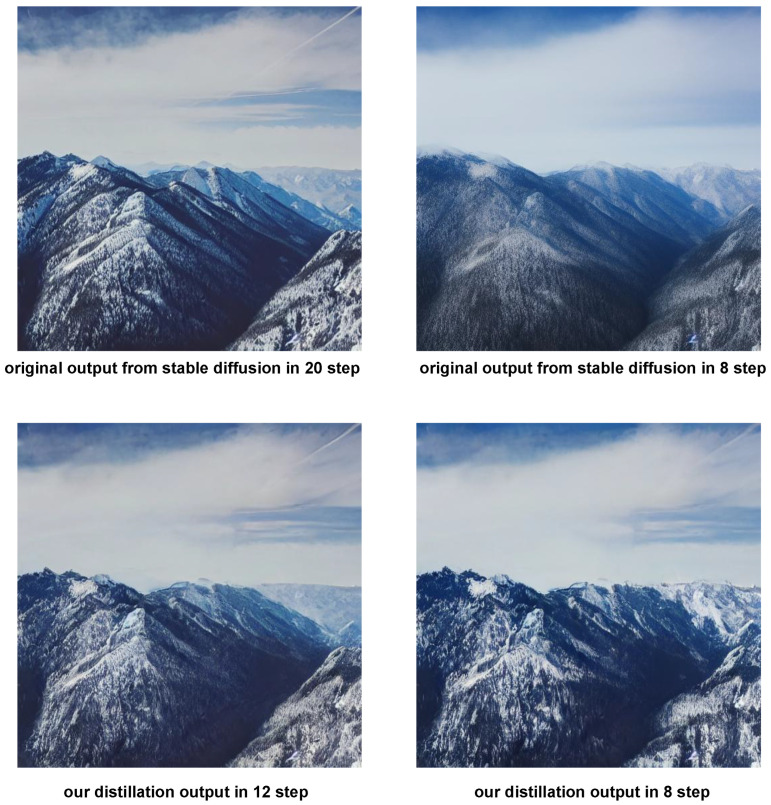
Output images from the original stable diffusion model and our novel distillation algorithm.

Subsequently, without any additional training, we compared the FID scores of our generation method using 7 + 1 steps of inference with those of various models [14,21] distilled on the COCO dataset and LAION dataset [38]. The results are provided in Table 5, with FID values and clip score [39] reported in respective papers. Visualized results are shown in Figure 6, Figure A1, Figure A2 and Figure A3.

**Table 5:** FID and CLIP Score comparisons for the baseline and direct distillation in 8-step inference. The FID and CLIP Score results of progressive distillation and SnapFusion are reported by the paper [3,40].

Solver	FID	Clip Score
Progressive distillation; 8-step inference with w = 4.0 in COCO	21.0	0.31
Progressive distillation; 8-step inference with w = 8.0 in COCO	30.0	0.32
Progressive and adversarial distillation; 8-step inference in COCO	22.3	0.26
Our distillation; 7 + 1-step inference with w = 7.0 in COCO	16.8	0.29
SnapFusion [40]; 8-step inference in LAION	24.2	0.30
Progressive distillation; 8-step inference with w = 8.0 in LAION	26	0.30
DPM++ solver; 8-step inference with Karras schedule in LAION	25.6	0.32
DPM solver; 8-step inference with Karras schedule in LAION	31.7	0.32
Our distillation; 7 + 1-step inference with w = 7.0 in LAION	24.4	0.30

Meanwhile, we also compare our distillation model with the latest distillation models [14,15,16,17,18,21] using adversarial loss in Table 6 to provide evidence about our mathematic proof in Section 3. As mentioned above, distillation algorithms using adversarial loss confront the diversity problem; the only exception is multi-student distillation which trains different student models to predict different image subsets to prevent the capacity problem. For diffusion distillation, the diversity problem of generative images mainly appears in large datasets, like COCO and LAION. We assume that this is because it relates to the capacity of the model, and it is not obvious [3,17,18,21] in small datasets like ImageNet (64 × 64) [41], where FID results for different models are also good. Meanwhile, our comparison mainly used the COCO dataset, since most of the distillation models are based on stable diffusion which originally trained on the LAION dataset; hence, some researchers only report FID results for the COCO dataset [17,18].

**Figure 7:**
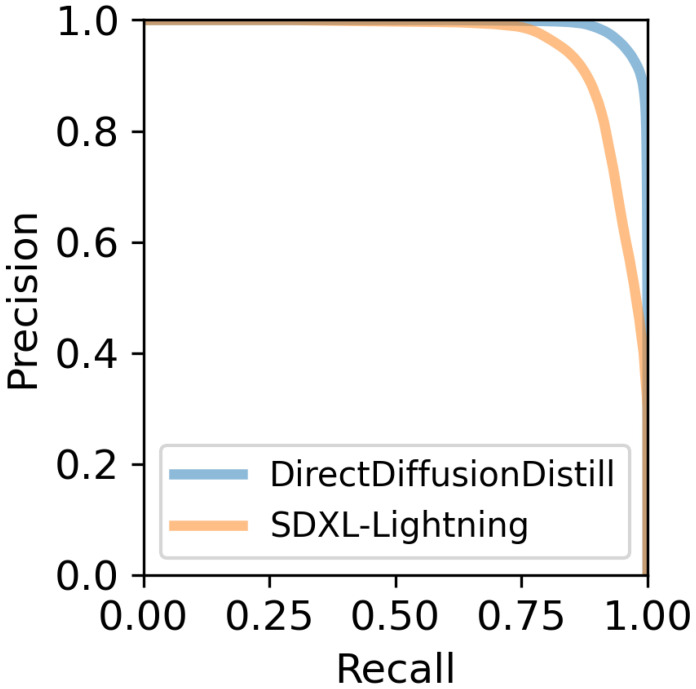
The comparison between direct distillation and adversarial progressive distillation in the standards of Precision and Recall for distributions.

To further provide evident proof that the FID bottleneck of progressive distillation is the diversity of generative images, which we mathematically prove in Section 3.3, we use Precision and Recall for Distribution (PRD) [42] to assess the generative result of the adversarial progressive distillation model and our proposed distillation model. Figure 7 shows that diversity problems are the main difference between the generative distribution of SDXL-Lightning and ground truth distribution since the lack of Recall means the ground truth cannot be shown completely in generated images. Meanwhile, even though the adversarial progressive distillation algorithm has SOTA implementation, SDXL-Lightning is renowned for generating high-resolution images and is the backbone of the assessing neural network proposed by RPD [42], Inception-V3, which means that the input of images will automatically resize to 299 × 299. Hence, we assume that the assessing network cannot completely capture the difference in image quality for high-resolution images generated by SDXL-Lightning and our distillation model.

## 5. Discussion

The proposed diffusion sampling process offers additional insights into the distillation step, which removes noise step by step during inference. The primary goal of distillation is to identify one critical step. Prior to this point, the primary task involves generating the image. After this point, the primary task is a simple denoising step. As a hierarchy-VAE, it is rational to assume that hidden variables in noisy regions generate outputs primarily containing low-frequency semantic information. In clean regions, the model primarily contributes to high-frequency details. These questions are used to guide the exploration of the network structure of stable diffusion. The primary element of each inference step is an attention block that employs a counter-intuitive design, in which the input of the value and key matrix is not a noisy image but a prompt embedding generated by CLIP, which uses noisy images as queries to fetch information from prompt embeddings to generate images [43]. Previous studies have shown that higher visual cortices in the human brain can be properly projected as features generated by CLIP and used in diffusion models, which assume that prompt embeddings mainly contain abstract and reasonable semantic information [44]. Our proposed distillation algorithm also demonstrates that using a lightweight network without involving any prompt embeddings can denoise the middle features directly, generating the final output and partly clarifying in which step the process becomes a denoising task. Prior to this step utilizing the proposed distillation model, this result allows the generation of images to mimic a translation that extracts semantic meaning from prompt embeddings via Gaussian noise. The substantial difference in both structure and computational resources between the original model and the distillation model can partly be seen as a non-strict ablation experiment. The main difference between the stable diffusion model in the clean and noisy regions demonstrates the redundancy of using stable diffusion for denoising.

**Figure 8:**
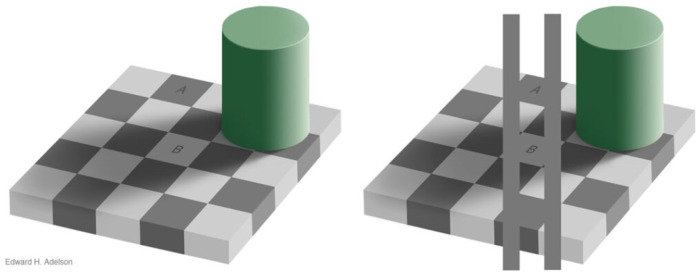
Output images from conventional stable diffusion and novel distillation algorithms.

The proposed generative model also has potential applications in neuroscience. A popular consciousness theory in computational neuroscience, the free energy principle (FEP) [45], uses Bayesian analysis to explain the function of consciousness. One renowned example is the checker shadow illusion shown in Figure 8, which claims that our brain does not naively store the absolute value of color but uses Bayesian inference to deduct hidden variables for light entering our eyes. However, vanilla Bayesian inference is inefficient and the variational method is more appropriate for real-world tasks. FEP uses ELBO in the AIGC area to describe the mechanisms of consciousness. However, using a generative model to construct hidden variables and make predictions encounters several issues commonly faced by reinforcement learning (RL). For example, the dark room problem [46] involves placing an animal in a dark room. With the guidance of minimized surprises described by free energy, which is represented by ELBO use in the AIGC area, the animal will stay in the dark room. One popular theory as to why animals choose not to remain in dark rooms is that the animal itself predicts that it should leave the dark room, which implies that FEP cannot be falsified as in RL. Meanwhile, previous studies have shown that evolution may prevent animals from using Bayesian inference to understand their environment [47,48]. Hence, investigating how GAN-like situations affect Bayesian inference remains an interesting problem.

## 6. Conclusions

This study proposes a novel distillation algorithm and a new time schedule, guided by an information bottleneck, which ensures a diverse variety of generative images by preventing accumulating generalization errors in the initial stages of the reverse process. Empirical knowledge that the diffusion process will converge in different steps prevents the need for the additional training of our distillation algorithm and allows for a 30–50% reduction in sample steps using our new schedule. We also demonstrate the relationship between the distillation of SD, a special HVAE, and GANs in the VIB framework. These results confirm that the proposed distillation model and time schedule are viable new strategies that could replace conventional diffusion in image generation algorithms. Our proposed distillation algorithm achieved better FID results compared with strong counterparts, including DPM++, progressive distillation, guided distillation, and adversarial diffusion distillation. The main reason for this result is that the proposed distillation ensured the diversity of generative images by precisely trading off the mutual information between the middle feature and the final output. Some of the distillation algorithms mentioned above can be enhanced by considering the information theory framework proposed in this paper. For example, adversarial distillation can use the original model to iteratively predict the middle feature while using the distillate model with adversarial training to predict the final result, which can ensure mutual information between the middle feature and the final result. In our proposed algorithm, we ensure generative diversity, without explicitly designing an improvement method to rectify the image quality, which could be considered the main direction of the future work. Meanwhile, whether our algorithm can cope with the possible diversity problem in different modalities needs further research.

## Figures and Tables

**Table 1:** A performance comparison between our time schedule and the Karras schedule, using 10,000 random captions from the COCO dataset.

Time Schedule	FID
Our improved time schedule with a DPM++ solver in 20 steps	16.64
The Karras time schedule with a DPM++ solver in 20 steps	17.36

**Table 2:** Parameters and MACs for various models.

Model	MACs (M)	Parameters (M)
UNet2DCondition from SD	67749	859
Direct distillation first stage	3954.1	57.5
Direct distillation second stage	3922.5	13.5

**Table 3:** A comparison of FID performance for the distillation model and the original diffusion model applied to the COCO validation set.

Time Schedule	FID
New schedule with DPM++ 2m solver in 20 steps	12.59
New schedule with distillation solver in 12+1 steps	14.15

**Table 4:** A comparison of FID performance for the distillation model and the original diffusion model applied to a sample of 10,000 random images from the COCO validation set.

Time Schedule	FID
DPM++ 2m solver in 12 steps with Karras	14.5
Direct distillation in 12 + 1 step with w = 7.0	14.9

**Table 6:** FID comparisons between direct distillation and recent popular distillation algorithms using adversarial loss. The FID results of LCM-SDXL, SDXL-Turbo, SDXL-Lightning, and DMD2 are reported by the paper [17]. The FID result of MSD is reported by the paper [18].

Solver	FID	Clip Score
LCM-SDXL in 4 steps in COCO	22.16	0.31
SDXL-Turbo in 4 steps in COCO	23.24	0.334
SDXL-Lightning in 4 steps in COCO	24.46	0.323
Progressive distillation in 8 steps with w = 4.0 in COCO	21.0	0.31
Progressive distillation in 8 steps with w = 8.0 in COCO	30.0	0.32
DMD2 in 4 steps in COCO	19.32	0.332
Our distillation in 7 + 1 steps with w = 7.0 in COCO	16.8	0.29
MSD: 4 students, ADM in COCO	8.20	0.30

## Data Availability

Data are contained within this article.

## Abbreviations

The following abbreviations are used in this manuscript:

VAE	Variational Auto Encoder
GAN	Generative Adversarial Network
DDPM	Denoising Diffusion Probabilistic Model
DPM-Solver	Diffusion Probabilistic Model Solver
DPM++-Solver	Diffusion Probabilistic Model ++ Solver
IB GAN	Information Bottleneck Generative Adversarial Network
VIB	Variational Information Bottleneck
FEP	Free Energy Principle
ELBO	Evidence Low Bounce
CLIP	Contrastive Language–Image Pre-Training
COCO Dataset	Common Objects in Context Dataset
FID	Fréchet Inception Distance
SD	Stable Diffusion
FLOPS	Floating Point Operations Per Second
MACS	Memory Access Cost Per second
ODE	Ordinary Differential Equation
SDE	Stochastic Differential Equation
SOTA	State of the Art
NAFNet	None-Linear Activation-Free Network
DPI	Data Process Inequality
KL Divergence	Kullback–Leibler Divergence

## Appendix A

Here we present comparisons of distillation results for the original generative model.
